# Efficacy of COVID-19 Treatments in Intensive Care Unit: A Systematic Review and Meta-Analysis of Randomized Controlled Trials

**DOI:** 10.1155/ccrp/2973795

**Published:** 2024-11-27

**Authors:** Mahmoud Alwakeel, Francois Abi Fadel, Abdelrahman Nanah, Yan Wang, Mohamed K. A. Awad, Fatima Abdeljaleel, Mohammed Obeidat, Talha Saleem, Saira Afzal, Dina Alayan, Mary Pat Harnegie, Xiaofeng Wang, Abhijit Duggal, Peng Zhang

**Affiliations:** ^1^Department of Pulmonary and Critical Care Medicine, Respiratory Institute, Cleveland Clinic, Cleveland, Ohio, USA; ^2^Department of Medicine, Cleveland Clinic Fairview Hospital, Cleveland, Ohio, USA; ^3^Department of Anesthesiology, Boston Medical Center, Boston, Massachusetts, USA; ^4^Department of Pulmonary, Critical Care and Allergy, University of Alabama, Birmingham, Alabama, USA; ^5^Department of Neurology, Cleveland Clinic Florida, Weston, Florida, USA; ^6^Department of Internal Medicine, Cleveland Clinic Florida, Cleveland, USA; ^7^Floyd D. Loop Alumni Library, Cleveland Clinic, Cleveland, Ohio, USA; ^8^Department of Qualitative Health Sciences, Cleveland Clinic, Cleveland, Ohio, USA

## Abstract

**Objectives:** Examining the cumulative evidence from randomized controlled trials (RCTs), evaluating the use of pharmacological agents for the treatment of COVID-19 infections in patients with critical illness.

**Data Sources:** Databases Medline, Embase, Web of Science, Scopus, CINAHL, and Cochrane.

Study Selection: Inclusion criteria were RCTs that enrolled patients with confirmed or suspected COVID-19 infection who are critically ill. Only RCTs that examined therapeutic agents against one another or no intervention, placebo, or standard of care, were included.

**Data Extraction:** Pairs of reviewers extracted data independently. Outcomes of interest included the overall reported mortality defined as either the ICU mortality, hospital mortality, mortality within 28 days or mortality within 90 days.

**Data Synthesis:** A total of 40 studies (11,613 patients) evaluated 50 therapeutic intervention arms divided into five main therapy categories; steroids, antiviral medications, immunomodulators, plasma therapies [intravenous immunoglobulins (IVIG), convalescent plasma and/or, therapeutic plasma exchange], and therapeutic anticoagulation. Immunomodulators was the only group with possible mortality benefit, risk ratio (RR) 0.83 (95% CI 0.73; 0.95), with nonsignificant heterogeneity (*I*^2^ = 8%, *p*=0.36). In contrast, the other therapy groups showed no significant impact on mortality, as indicated by their respective pooled RRs: steroids [RR 0.91 (95% CI 0.82; 1.01), *I*^2^ = 31%], antiviral medications [RR 1.11 (95% CI 0.82; 1.49), *I*^2^ = 57%], plasma therapies [RR 0.77 (95% CI 0.58; 1.01), *I*^2^ = 36%], and anticoagulation [RR 1.06 (95% CI 0.95; 1.18), *I*^2^ = 0%].

**Conclusions:** This meta-analysis highlights both the heterogeneity and a lack of benefit from therapies evaluated during the COVID-19 pandemic. Many of the RCTs were developed based on limited observational data. Future RCTs investigating pharmaceutical interventions in critically ill patients during pandemics need to be designed based on better evidence.

## 1. Introduction

Since the beginning of the COVID-19 pandemic more than 8000 randomized controlled trials (RCTs), in different stages of progression and completion have been conducted to identify a therapy to achieve survival benefit in COVID-19-associated infection [1]. Over the last 3 years more than 500 meta-analyses have been published to summarize and develop an evidence base for the efficacy of pharmacologic agents for the treatment of COVID-19 infections [2]. The desire for effective therapy is even more heightened in the intensive care unit (ICU) settings where the majority of mortality from the pandemic occurred [3].

Unfortunately, most therapeutic RCTs for COVID-19 have enrolled patients with varying degrees of severity of disease and need for oxygen support, and consequently have had to grapple with significant heterogeneity in the included populations [4]. Evidence that looked exclusively in the ICU is limited to early case series from single centers or from studies with inherent biases, suboptimal study designs and inappropriate statistical analysis [5]. These initial studies were used to inform treatment in many countries, despite these significant limitations [6]. Subsequent well-designed studies developed by research consortia globally (REMAP [7] and ACTIV [8]) mostly reported outcomes in ICU patients as subgroups. Even though these results were compelling, the issue of type I errors in those groups remains a concern [9, 10].

For that reason, a systematic review and meta-analysis was performed examining the cumulative evidence from RCTs, which have studied pharmacologic agents exclusively in the ICU setting and how those results would differ when compared to the larger studies performed in all hospitalized adults, which informed the majority of treatment decisions for patients with critical illness in the later phases of the pandemic.

## 2. Methods

This systematic review and meta-analysis follow the preferred reporting items for systematic reviews and meta-analyses (PRISMA) checklist (Supporting Figure 11) [11]. The protocol was registered with the International prospective register of systematic reviews (PROSPERO# CRD42021250583https://www.crd.york.ac.uk/prospero/display_record.php?RecordID=250583) [12].

### 2.1. Eligibility Criteria

#### 2.1.1. Studies and Participants

Studies included were RCTs that included patients with confirmed or suspected COVID-19 infection who were critically ill. Critical illness was defined as any patient admitted to an adult ICU or area of the hospital where critically ill patients routinely received treatment, patients receiving invasive or noninvasive mechanical ventilation (MV), patients receiving continuous intravenous vasoactive medications or other criteria with justification presented in the individual study to designate patients as critically ill. Only trials published in English language were included.

#### 2.1.2. Interventions and Comparisons

Only RCTs that examined therapeutic agents against one another or no intervention, placebo, or standard of care were included. Exclusion criteria were RCTs evaluating vaccinations, nutrition, vitamins, herbal, or alternative medicines, and nonmedication-related supportive interventions such as proning.

### 2.2. Outcomes

The outcome of interest for this study was the overall reported mortality defined as either the ICU mortality, hospital mortality, mortality within 28 days, or mortality within 90 days.

### 2.3. Information Source

A systematic search of existing, relevant literature was performed by the authors, including an experienced medical information specialist, in the databases: Medline (via Ovid SP), Embase (via Ovid SP), Web of Science (via Clarivate Analytics), Scopus (via Elsevier), CINAHL (via EBSCOhost), and Cochrane (via Wiley). The databases were searched from Jan 1^st^, 2019, till April 21^st^, 2021 and updated twice on Sept 30^th^, 2021, and April 28^th^, 2023. Keywords: COVID, coronavirus, coronavirus infection, SARS, critical care, intensive care, artificial respiration, MV, vasoconstrictor agent, and RCTs. A comprehensive description of the search strategy is included in the supporting information file. The articles were imported from Endnote (version X8.2 for Windows; 2018 Clarivate, Philadelphia, USA) to Covidence systematic review software (Veritas Health Innovation, Melbourne, Australia. Available at https://www.covidence.org) and checked for duplicates.

### 2.4. Study Selection

Using the systematic review software, Covidence, pairs of reviewers, following training and standardization exercises, independently screened all titles and abstracts, followed by full texts of RCT that were identified as potentially eligible. If a conflict existed, a third independent reviewer resolved it.

### 2.5. Data Collection

For each eligible trial, pairs of reviewers extracted data independently using a standardized data collection form on Microsoft Excel 2013. Reviewers collected information on trial characteristics (trial PubMed reference number, registration number, study country, sample size), patient characteristics (age, sex, and the number of patients on MV), and mortality outcomes of interest; the number of participants analyzed and the number of participants who experienced an event for dichotomous outcomes. Discrepancies were resolved by an independent third reviewer.

### 2.6. Risk of Bias Within Individual Studies and Publication Bias Assessment

Two independent reviewers assessed the risk of bias for each study based on the criteria outlined in Version two of the Cochrane risk-of-bias tool for randomized trials [13]. These criteria included random sequence generation, blinding of participants and personnel, blinding of outcome assessment, allocation concealment, incomplete outcome data, selective reporting, and other potential sources of bias. For each criterion, the risk of bias was categorized as low, high, or unclear. Analyses of the data and figures were computed using the RevMan 5.4.1 software. Disagreements were solved by discussion between the reviewers; if necessary, a consensus was sought through consulting a third reviewer. To assess the potential presence of publication bias in the analysis, a funnel plot was utilized, a standard graphical tool for visualizing the symmetry of effect size estimates. To further test the symmetry of the funnel plot, in addition to the visual examination, Egger's regression test was conducted.

### 2.7. Data Synthesis and Statistical Analysis

In the meta-analysis, each node represents a treatment medication, regardless of the dose or duration of administration. For studies involving more than a therapeutic intervention arm like different dosages from the drug or different combination, each arm was treated as a separate group compared to the same number of the control arm. The studies were divided into a total of five treatment groups depending on the intervention drug category; steroids, antiviral medications, plasma therapies (IVIG, convalescent plasma and/or therapeutic plasma exchange), immunomodulators, and agents targeting therapeutic anticoagulation. This classification was developed based on the author's consensus. Treatment effects were expressed as risk ratios (RRs) for mortality. Pooled RR was calculated using random effects models, as clinical heterogeneity was expected. Statistical heterogeneity among the studies' effects was investigated using the *I*^2^ statistic. All statistical analyses were performed using R programming language version 4.2.2 (R Foundation for Statistical Computing, Vienna, Austria. URL https://www.R-project.org/).

## 3. Results

**Figure 1:**
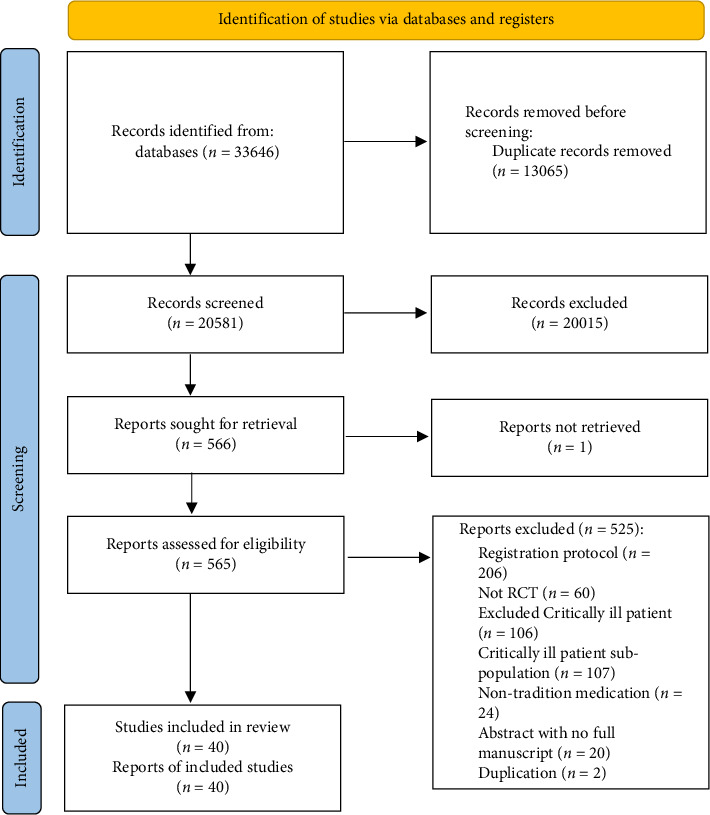
Flow diagram showing the identification of eligible trials and participating trials.

After removing duplicates, 20,581 title/abstracts were screened. Subsequently, 565 studies were assessed for full-text eligibility. 40 studies (11,613 patients) with a total of 50 therapeutic intervention arms met inclusion criteria (Figure 1). An overview of the characteristics of the included studies is provided in Table 1.

**Figure 2:**
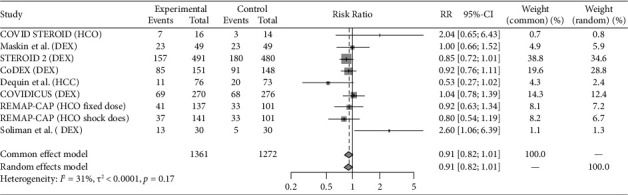
The pooled analysis for the mortality in steroid group forest plot of the patients received steroid medication as intervention versus the control group. The pooled risk ratio (RR) of overall mortality.

**Figure 3:**
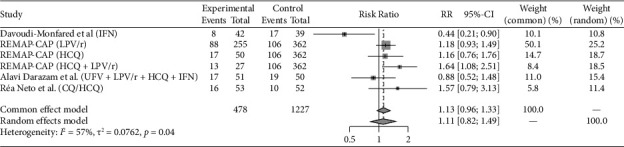
The pooled analysis for the mortality in antiviral group forest plot of the patients received antiviral medication as intervention versus the control group. The pooled risk ratio (RR) of overall mortality.

**Figure 4:**
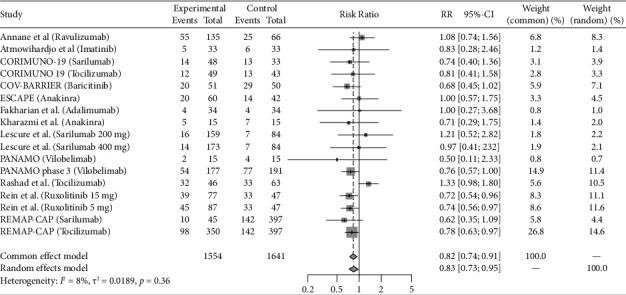
The pooled analysis for the mortality in immunomodulators group forest plot of the patients received immunomodulators medication as intervention versus the control group. The pooled risk ratio (RR) of overall mortality.

**Figure 5:**
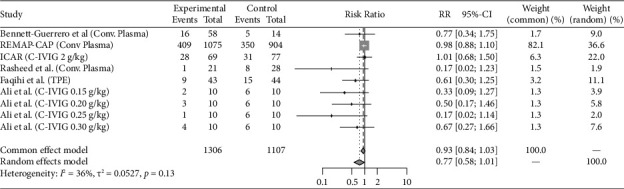
The pooled analysis for the mortality in plasma therapies group forest plot of the patients received plasma therapies medication as intervention versus the control group. The pooled risk ratio (RR) of overall mortality.

**Figure 6:**
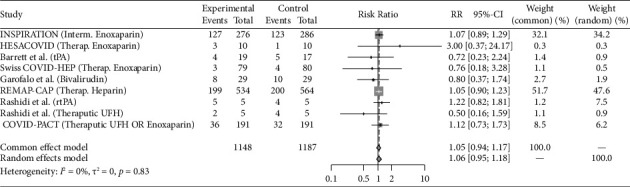
The pooled analysis for the mortality in anti-coagulation group forest plot of the patients received anticoagulation medication as intervention versus the control group. The pooled risk ratio (RR) of overall mortality.

Eight studies (REMAP-CAP steroid 2020 [14]; CoDEX 2020 [15]; Dequin et al. 2020 [16]; COVID STEROID 1 2021 [17], COVID STEROID 2 2021 [18], Maskin et al. 2021 [19], COVIDICUS 2022 [20], Soliman et al. 2022 [21]) with total 9 therapeutic arms (Hydrocortisone *n* = 4, Dexamethasone *n* = 4, Methylprednisolone *n* = 1) assessed the use of Steroids exclusively in critically ill patients. Antivirals were studied in 4 studies (Davoudi-Monfared et al. 2020 [22]; REMAP-CAP antiviral 2021 [23]; Réa-Neto et al. 2021 [24]; Alavi Darazam et al. 2021 [25]) with a total 6 therapeutic arms (Lopinavir-Ritonavir *n* = 1, Hydroxychloroquine, or Chloroquine *n* = 2, Interferon-*β*1a *n* = 1, Lopinavir-Ritonavir + Hydroxychloroquine *n* = 1, Lopinavir/Ritonavir + Hydroxychloroquine + Interferon-*β*1a + Umifenovir *n* = 1). Fourteen studies evaluated immunomodulators in critically ill patients (PANAMO 2020 [26]; Fakharian et al. 2021 [27]; Rashad et al. 2021 [28]; REMAP-CAP immunomodulator 2021 [29], COV-BARRIER 2021 [30], Lescure et al. 2021 [31], PANAMO 2022 [32], Atmowihardjo et al. 2022 [33], CORIMUNO-19 TOCI-2 2022 [34], CORIMUNO-19 SARI-2 2022 [34], ESCAPE 2022 [35], Kharazmi et al. 2022 [36], Rein et al. 2022 [37], Annane et al. 2023 [16]) with a total of 17 therapeutic arms (Tocilizumab *n* = 3, Sarilumab *n* = 4, Adalimumab *n* = 1, Vilobelimab *n* = 2, Ravulizumab *n* = 1, Imatinib *n* = 1, Baricitinib *n* = 1, Anakinra *n* = 2, Ruxolitinib *n* = 2). Plasma-related interventions were studied in 6 RCTs (Rasheed et al. 2020 [38]; Faqihi et al. 2021 [39]; Ali et al. 2021 [39]; Bennett-Guerrero et al. 2021 [40], REMAP-CAP 2021 [41], ICAR 2022 [42]) with a total of 9 therapeutic arms (Therapeutic plasma exchange (TPE) *n* = 2, convalescent plasma *n* = 7). Eight studies evaluated anticoagulation exclusively in critically ill patients (HESACOVID 2020 [43]; INSPIRATION 2021 [44]; REMAP-CAP 2021 [45], Barrett et al. 2021 [46], Rashidi et al. 2022 [47], Swiss COVID-HEP 2022 [48], COVID-PACT 2022 [49], Garofalo et al. 2022 [50]) with total 9 therapeutic arms (Therapeutic anticoagulation by heparin, enoxaparin or Bivalirudin *n* = 7, Alteplase *n* = 2). The control groups varied between placebo, the standard of care, and different medication regimens summarized in Table 1. All the included studies are published. COVID STEROID-1 [17] was terminated early because of evidence from other studies indicating the benefit of corticosteroids in severe COVID-19. Also, Maskin et al. [19] study terminated early due to low enrollment rate. Studies that incorporated two or more different therapy categories within the same intervention arm were excluded, as the efficacy could not be attributed specifically to one category [51–53]. Outcomes reported for the individual studies included in this analysis are reported in Table 2. Figures 2, 3, 4, 5, and 6 summarize the reported overall mortality outcomes and the estimated pooled effect. For a detailed presentation of the pooled effects for ICU, hospital, 28 days, and 90 days mortality, forest plots are provided in the Supporting Figures 6–10.

### 3.1. Steroid

Among the included studies, 32.5% (443/1361) of the intervention cohorts and 35.8% (456/1272) of the control cohorts experienced mortality. The pooled estimated RR using a random-effects model was 0.91 (95% CI 0.82; 1.01), with nonsignificant heterogeneity (*I*^2^ = 31%, *p*=0.17).

### 3.2. Antiviral

Approximately, one-third of participants in both cohorts died by the conclusion of the study period. In the intervention cohort, the mortality rate was 33.3% (159 out of 478), while it was 29.7% (364 out of 1227) in the control cohort. The pooled estimated RR using a random-effects model was 1.11 (95% CI 0.82; 1.49), and there was significant heterogeneity (*I*^2^ = 57%, *p*=0.04).

### 3.3. Immunomodulators

Among the included studies, 28.6% (445/1554) of the intervention cohorts and 35.9% (589/1641) of the control cohorts experienced mortality. The pooled estimated RR using a random-effects model was 0.83 (95% CI 0.73; 0.95), with nonsignificant heterogeneity (*I*^2^ = 8%, *p*=0.36).

### 3.4. Plasma Therapies

Among the included studies, 36.2% (473/1306) of the intervention cohorts and 39.1% (433/1107) of the control cohorts experienced mortality. The pooled estimated RR using a random-effects model was 0.77 (95% CI 0.58; 1.01), with nonsignificant heterogeneity (*I*^2^ = 36%, *p*=0.13).

### 3.5. Anticoagulation

Among the included studies, 33.7% (387/1148) of the intervention cohorts and 32.3% (383/1187) of the control cohorts experienced mortality. The pooled estimated RR using a random-effects model was 1.06 (95% CI 0.95; 1.18), with nonsignificant heterogeneity (*I*^2^ = 0%, *p*=0.83).

### 3.6. Risk of Bias

**Figure 7:**
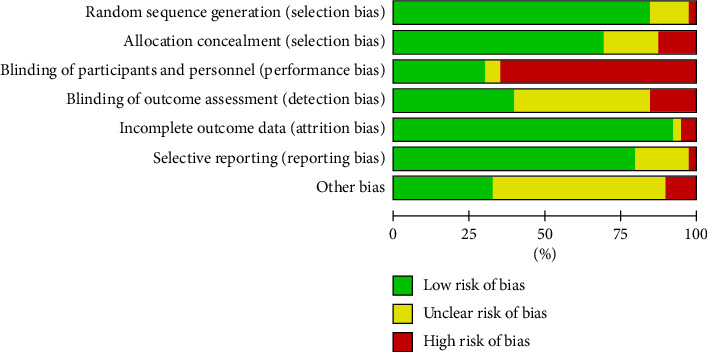
Summary of Cochrane risk of bias assessment of the included trials.

**Figure 8:**
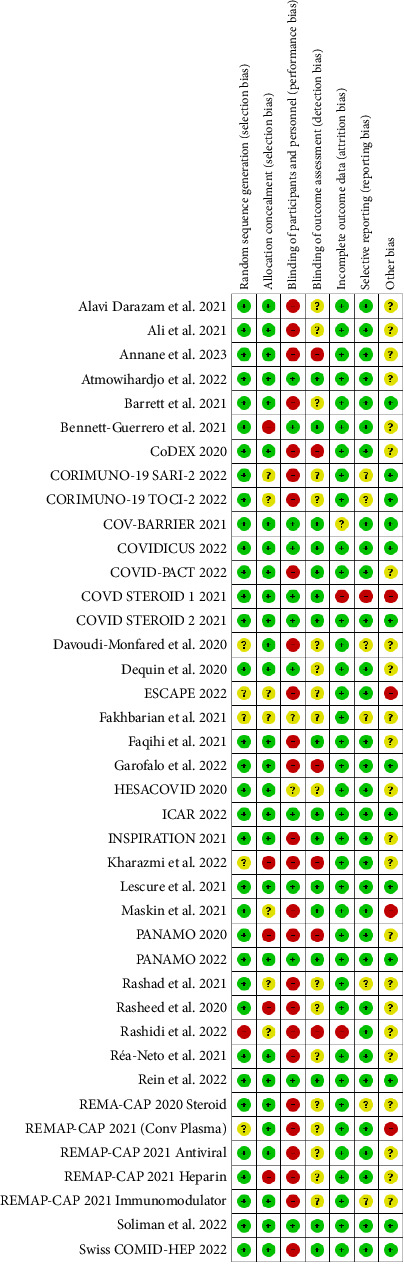
The detailed Cochrane risk of bias assessment of the included trials data could be extracted from tables and figures in the manuscript.

Of the RCTs, 85% (34 out of 40) reported adequate generation of the random sequence, 73% (29/40) of the RCTs reported adequate allocation sequence concealment, and 40% (16/40) of the included RCTs reported adequate blinding of the outcome assessors. A graph of the risk of bias and the assessment of each RCT are shown in Figures 7 and 8. Assessing publication bias posed challenges in this analysis, primarily due to the relatively low number of available publications. Upon visual inspection of the funnel plot, no apparent asymmetry was observed in the steroid and immunomodulators groups. The results of Egger's regression test confirmed this, yielding *p* values of 0.27 and 0.89, respectively. In contrast, noticeable asymmetry was detected in the antiviral, plasma therapies, and anticoagulations groups. However, Egger's regression test reached statistical significance only in the plasma therapies group (*p* < 0.01), while remaining nonsignificant in the antiviral (*p*=0.27) and anticoagulation groups (*p*=0.48). Funnel plot diagrams and Egger's regression test results were summarized in Supporting Diagrams 1–5.

## 4. Discussion

This systematic review and meta-analysis of COVID-19-related therapeutic interventions exclusively in patients with critical illness revealed that many of the trials that focused on patients with critical illness were usually underpowered, from single centers or health care systems and were disproportionately from the early parts of the pandemic. Most of the large international trials did not focus adequately on outcomes related to patients with critical illness exclusively and only reported on this population as a subgroup. This systematic review also highlights that many unproven therapies including different antivirals and convalescent plasma were used in this vulnerable and highly tenuous population despite limited scientific rationale. These decisions were likely driven by fact that many health care systems globally were overwhelmed and a sentiment of using any potential therapy as a rescue intervention was justified by clinicians and researchers. This systematic review also highlights that in many cases this led to multiple interventions being introduced for many patients, making the analysis of any effectiveness studies for specific medications almost impossible. But perhaps most importantly this meta-analysis highlights the significant heterogeneity in the quality, included populations and the outcomes reported in these studies. This heterogeneity is potentially a contributing factor in the disparate results seen in many of these studies.

### 4.1. Steroids

Similar to the findings reported by the WHO Rapid Evidence Appraisal for COVID-19 Therapies (REACT) Working Group meta-analysis [54] (September 2020); using random-effects modeling [OR 0.70 (95% CI, 0.48–1.01; *p*=0.053)], this present study did not identify a survival advantage associated with the use of steroids. The REACT analysis included only two studies that exclusively focused on patients with critical illness, in contrast to the approach taken by the British Medical Journal's (BMJ) living network meta-analysis [55]. This study has reported a favorable pooled effect of systematic steroids on COVID-19 patient mortality regardless of the severity, (OR 0.80, 95% CI 0.65–0.94). Unfortunately, the BMJ study did not conduct subgroup analyses, making direct comparisons with present study results challenging. It is important to highlight that the landmark study, RECOVERY: COVID-19 Dexamethasone [56] was excluded from the analysis. We made this decision because the patients with critical illness were only reported as a subgroup of this study. While the RECOVERY study did report a reduced risk of 28-day mortality for patients on MV compared to those not on MV (with a relative risk of 0.64 and a 95% CI of 0.51–0.81), it's crucial to acknowledge that the definition of patients with critical illness extends beyond those solely on MV. Therefore, generalizing the results to all critically ill patients not on MV would be overly restrictive.

### 4.2. Antivirals

Current study findings align with the limited evidence regarding the effectiveness of antiviral treatments in critically ill COVID-19 patients. Lee et al. [57] found no significant impact of Remdesivir on mortality for MV patients (RR 1.08, 95% CI, 0.88–1.31). Consistent results have been observed in a meta-analysis that included RCTs with critically ill patients only. In a recent Living Systematic Review and Meta-analysis conducted by Kaka et al. [58], (March 2022) no difference in mortality was observed (absolute risk difference −0.7% [95% CI, −2.4%–1.0%]. Also, in the living Network meta-analysis by BMJ [55] the OR of mortality was not significant in Remdesivir studies (OR 0.91, 0.73–1.11) and lopinavir-ritonavir studies (1.06, 0.88–1.28). Although none of the Remdesivir RCTs met this study inclusion, the existing evidence from the previous meta-analysis supports the nonsignificant effect for antiviral drugs in critically ill COVID patients. The DisCoVeRy trial [59] (September 2021), which examined the efficacy of Remdesivir could not find any mortality benefit at 28 days, OR 0.93 (95% CI, 0.57–1.52). Also, the largest trial that examined four different antiviral medications, the Solidarity trial [60] (February 2021), Remdesivir, Hydroxychloroquine, Lopinavir, and interferon beta-1a, had little or no effect on hospitalized patients with COVID-19, as indicated by overall mortality. Also, they calculated the pooled effect of the available trials that examined Remdesivir, Hydroxychloroquine, and Lopinavir and none of them were significant, RR 0.91 (95% CI, 0.79–1.05), 1.09 (95% CI, 0.98–1.21), 1.01 (95% CI, 0.91–1.13), respectively. RECOVERY [61] trial group reported a similar conclusion regarding Lopinavir-Ritonavir, 28-day mortality (RR 1.03, 95% CI, 0.91–1.17) that remained nonsignificant even after stratification for a critically ill patient.

### 4.3. Immunomodulators

This study outcome closely mirror the findings of the WHO REACT [62] group, indicating that the use of IL-6 antagonists such as Tocilizumab and Sarilumab in COVID-19 patients may reduce 28-day mortality, with an OR of 0.86 and a 95% CI of 0.79–0.95 (*p*=0.003). A subgroup analysis failed to show a significant benefit for patients on advanced respiratory or cardiovascular support at the time of randomization. There is no available meta-analysis for Vilobelimab or anti-TNF in subgroups of patients with critical illness at the time of RCT enrollment. RCTs excluded due to subgroup analyses focusing on patients with critical illness did not demonstrate a significant mortality benefit. For instance, in the RECOVERY [63] examining Tocilizumab, the RR for 28-day mortality was as follows when stratified by levels of respiratory support: 0.81 (0.67–0.99) for no ventilator support, 0.86 (0.74–1.00) for noninvasive ventilation (NIV), and 0.93 (0.74–1.18) for MV.

### 4.4. Plasma Therapies

Few meta-analyses examined the TPE in patients with critical COVID-19. Wardhani et al. [64] (Dec 2021) and Qin et al. [65] (Jun 2022) concluded that the TPE in patients with critical illness subgroup decreased all cause-mortality; OR 0.21 (95% CI: 0.05–0.85) and RR 0.41 (95% CI 0.24–0.69), respectively. Both studies included only one RCT in addition to five or fewer retrospective studies, which could affect the accuracy of the result and weaken the level of evidence. Two meta-analyses examined the efficacy of convalescent plasma on COVID-19 patients and conducted a subgroup analysis on patients with critical illness. Axfors et al. [66] collaborative meta-analyses (Nov 2021) reported that convalescent plasma has not added a mortality benefit for patients with critical illness (2 trials with 2315 patients); RR 0.97 (0.65–1.44), and the same for all severity patients (33 trials; 16,477 patients); 0.97 (95% CI [0.92–1.02]). The same thing was reported by Marcec et al. [67]; RR for all-cause mortality in patients with critical illness was not significant; RR 86 (0.45–1.57). Although this study results are similar to the previous two studies it is important to clarify that this meta-analysis combined convalescent plasma and TPE as one group as we thought it is more continent for categorization. Removing the TPE study from this analysis did not affect the statistical significance. This study excluded the RECOVERY [68] trial because the patients with critical illness were a subgroup. The study could not find a benefit for the convalescent plasma in COVID-19 patients regardless of the severity and on subgroup patients on MV; (RR 1.00, 95% CI 0.93–1.07) and 0.86 (0.60–1.25), respectively.

### 4.5. Anticoagulation

This meta-analysis showed that escalated doses of anticoagulation in patients with critical COVID-19 did not have a significant benefit compared to a standard of care prophylaxis regimen, which is similar to that reported by Ortega-Paz et al. [69] (Sep 2022) meta-analysis. The author included Seven trials (5154 patients) Compared to standard-dose prophylactic anticoagulation, escalated-dose prophylactic anticoagulation was not associated with a reduction of all-cause mortality (17.8% vs. 18.6%; RR 0.96 [CI95%, 0.78–1.18]). Subgroup analysis for patients with critical illness (4 studies) was still nonsignificant; 1.03 (0.91–1.18). The author's study included the three big trials, which this study included in addition to the Perepu et al. [70] trial, which excluded in this study due to the patients with critical illness being a subgroup. Perepu et al. study had 107 patients admitted to an ICU at the time of enrollment and an intermediate dose of enoxaparin compared to a standard prophylactic dose did not lead to any improvement in all-cause mortality (HR 0.47; 95% CI, 0.19–1.15).

This study has multiple strengths, including the narrow and tight inclusion criteria to help reach a more robust and precise estimate of the currently available evidence for patients with critical COVID-19 specifically and try to minimize the heterogeneity in the inclusion criteria between the included studies. But those strong points were a source of limitation for this study since they led to few included studies, so a pooled summary could not be synthesized from all the included studies for each outcome examined, due to heterogeneity in the studies' outcomes reporting. These all highlight the need for well-designed RCTs specifically including patients with critical illness only with relevant clinical outcomes to be examined. Furthermore, only studies published in English were included. This may have limited the scope of the analysis and prevented the consideration of important findings reported in non-English publications. However, it is believed that the majority of relevant studies on COVID-19 have been published in English, and efforts were made to ensure that the search strategy was comprehensive and included multiple databases to mitigate this limitation. In future studies, exploring the sources of heterogeneity through meta-regression and conducting a network meta-analysis could provide deeper insights.

## 5. Conclusion

This meta-analysis highlights the heterogeneity in the available evidence coming from RCTs investigating different COVID-19 treatments in patients with critical illness. This systematic review and meta-analysis highlight the need to be a more robust and well-thought-out process to study patients with critical illness in future pandemics. Patients with critical illness are responsible for disproportionately high morbidity and mortality in viral pandemics, and decisions related to therapeutic interventions should not be based only on the analysis of subgroups, underpowered studies, and/or observational study designs. Critical care societies and researchers need to invest in more robust therapeutic options and appropriate study designs with adequate power for any future pandemic-related research.

## Figures and Tables

**Table 1:** Characteristics of included trials.

Group/Author	Protocol registration	Blinding	Country	Other inclusion criteria than critically ill COVID-19 patients^1^	No. of pts.	Intervention	Control	Primary outcome
*Steroids*								
REMAP-CAP steroid 2020	NCT02735707	Open-label	MCa		384	Fixed-dose HCO (50 mg or 100 mg every 6 h)	SOC	Respiratory and cardiovascular organ support–free days up to day 21
						Shock dependent HCO (50 mg every 6 h when shock was clinically evident)	SOC	
CoDEX 2020	NCT04327401	Open-label	Brazil	Receiving IMV within 48 h	299	DEX 20 mg IV for 5 days then 10 mg for 5 days	SOC	Ventilator-free days during the first 28 days
Dequin et al. 2020	NCT02517489	Double-blind	France	Within 24 h of the onset of the first severity criterion or 48 h for patients referred from another hospital	149	HCO 200 mg/day infusion	Placebo	Treatment failure on day 21^2^
COVID STEROID 1 2021	NCT04348305	Double-blind	Denmark		30	HCO 200 mg	Placebo	Days alive without the use of life support at day 28
COVID STEROID 2 2021	NCT04509973	Double-blind	MCb		971	DEX 12 mg/day for 10 days	DEX 6 mg/day for 10 days	Days alive without the use of life support at day 28
Maskin et al. 2021	NCT04395105	Open-label	Argentina	IMV for less than 72 h	98	DEX 16 mg IV for 5 days then 6 mg for 5 days	DEX 6 mg for 10 days	Ventilator-free days at day 28
COVIDICUS 2022	NCT04344730	Open-label	France	Within 48 h of ICU admission	546	DEX 20 mg IV for 5 days then 10 mg for 5 days	DEX 6 mg for 10 days OR placebo	Time to IMV
Soliman et al. 2022	NCT04909918	Double-blind	Egypt		60	DEX 8 mg/day for 7 days	MP 1 mg/kg/day for 7 days	NLR improvement in first 7 days

*Antivirals*								
Davoudi-Monfared et al. 2020	IRCT20100228003449N28	Open-label	Iran		81	IFN	SOC	Time to reach the clinical response
REMAP-CAP antiviral 2021	NCT02735707	Open-label	MCc		694	LPV/r	SOC	Respiratory and cardiovascular organ support–free days and in-hospital mortality
						HCQ	SOC	
						LPV/r + HCQ	SOC	
Alavi darazam et al. 2021	NCT04350684	Open-label	Iran	Symptoms ≤14 days	101	LPV/r + HCQ + IFN + UFV	LPV/r + HCQ + IFN	Time to clinical improvement
Réa-Neto et al. 2021	NCT04420247	Open-label	Brazil		105	HCQ or CQ	SOC	Clinical status of patients measured on day 14 with a 9-point ordinal scale

*Immunomodulators*								
PANAMO 2020	NCT04333420	Open-label	Netherlands		30	Vilobelimab	SOC	Percentage change in PaO2/FiO2 at baseline and day 5
Fakharian et al. 2021	IRCT20151227025726N23	Open-label	Iran		68	Adalimumab	SOC	Requiring IMV, admission to ICU, and the rate of mortality
Rashad et al. 2021	CT04519385 (not published)	Open-label	Egypt	Two positive tests of the following: (CRP > 10 g/L, lymphocytes < 600/mm3, D-dimer > 500 ng/mL, ferritin > 500 ng/mL)	109	Tocilizumab	Pulse DEX 4 mg/kg/day IV for 3 days, followed by 8 mg/day for 10 days	Time to failure (death within 14 days from ICU admission)
REMAP-CAP immunomodulator 2021	NCT02735707	Open-label	MCd		865	Tocilizumab	SOC	Number of respiratory and cardiovascular organ support–free days up to day 21
						Sarilumab	SOC	
COV-BARRIER 2021	NCT04421027	Double-blind	MCg		101	Baricitinib	Placebo	All cause mortality
Lescure et al. 2021	NCT04327388	Double-blind	MCh	Within 2 weeks of starting symptoms	416	Sarilumab 400 mg	Placebo	Clinical improvement (seven-point ordinal scale)
						Sarilumab 200 mg	Placebo	
PANAMO 2022	NCT04333420	Double-blind	MCe	Within 48 h of randomization	368	Vilobelimab	SOC	Mortality at 28 days
Atmowihardjo et al. 2022	NCT04794088	Double-blind	Netherlands		66	Imatinib	Placebo	Change in extravascular lung water between days 1 & 4
CORIMUNO-19 TOCI-2 2022	NCT04331808	Open-label	French		92	Tocilizumab	SOC	WHO-CPS score improvement
CORIMUNO-19 SARI-2 2022	NCT04331808	Open-label	French		81	Sarilumab	SOC	WHO-CPS score improvement
ESCAPE 2022	NCT04339712	Open-label	Greece		102	Anakinra	Tocilizumab	SOFA score or P/F ratio improvement
Kharazmi et al. 2022	IRCT20120703010178N20	Open-label	Iran		30	Anakinra	SOC	Need for IMV
Rein et al. 2022	NCT04377620	Double-blind	US & Russia	Within 3 weeks of starting symptoms	211	Ruxolitinib 15 mg twice daily	Placebo	28-day mortality
						Ruxolitinib 5 mg twice daily	Placebo	
Annane et al. 2023	NCT04369469	Open-label	MCf	Within 3 days of illness	201	Ravulizumab	SOC	All cause mortality

*Plasma therapies*								
Rasheed et al. 2020	BKH-CT-012 (not published)	NA	Iraq	First 3 days in ICU	49	Convalescent plasma	SOC	Safety profile
Faqihi et al. 2021	ISRCTN21363594	Open-label	Saudi Arabia	First 48 h of meeting the criteria for life-threatening COVID-19	87	Therapeutic plasma exchange	SOC	35-day mortality and safety profile
Ali et al. 2021	NCT04521309	Single-blinded (patient)	Pakistan		50	IVIG 0.15 g/kg	SOC	28-day mortality, patient's clinical status on ordinal scale and Horowitz index
						IVIG 0.20 g/kg	SOC	
						IVIG 0.25 g/kg	SOC	
						IVIG 0.30 g/kg	SOC	
Bennett-Guerrero et al. 2021	NCT04344535	Double-blind	USA		74	Convalescent plasma	Standard plasma	Ventilator-free days from randomization to day 28
REMAP-CAP **(**Conv. plasma) 2021	NCT02735707	Open-label	MCi		1987	Convalescent plasma	SOC	Respiratory and cardiovascular organ support–free days up to day 21
ICAR 2022	NCT04350580	Double-blind	France	Within 72 h of IMV	146	IVIG 2 g/kg	Placebo	Ventilation-free days by day 28

*Anticoagulations*								
HESACOVID 2020	REBEC RBR-949z6v	Open-label	Brazil		20	Enoxaparin 1 mg/kg BID	Standard anticoagulant thromboprophylaxis	Variation in PaO2/FiO2 at baseline, 7, and 14 days after randomization
INSPIRATION 2021	NCT04486508	Open-label	Iran	Within 7 days of the index hospitalization	562	Enoxaparin 1 mg/kg daily	Enoxaparin, 40 mg daily	Composite outcome3
REMAP-CAP 2021	NCT02735707	Open-label	MCj		1098	Therapeutic-dose of heparin	Standard anticoagulant thromboprophylaxis	Organ support–free days
Barrett et al. 2021	NCT04357730	Open-label	USA	IMV less than 11 days	36	rtPA	SOC	P/F ratio improvement in 48 h
Rashidi et al. 2022	IR.TBZMED.REC.1399.127	Open-label	Iran	Within 5 days of hospitalization	15	rtPA	SOC	P/F ratio improvement in 48 h
						Therapeutic UFH	SOC	
Swiss COVID-HEP 2022	NCT04345848	Open-label	Switzerland	Within 48 h of hospital admission	159	Enoxaparin 1 mg/kg BID OR therapeutic UFH	SOC	Composite of 30-day VTE, arterial thrombosis, DIC, and mortality
COVID-PACT 2022	NCT04409834	Open-label	USA	With 96 h of critical illness	382	Enoxaparin 1 mg/kg BID OR therapeutic UFH	SOC	Composite of venous and arterial thrombotic events
Garofalo et al. 2022	NCT05334654	Open-label	Italy	Within 24 h of randomization	48	Bivalirudin	SOC	IMV duration

Abbreviations: CQ, Chloroquine; DEX, dexamethasone; DIC, disseminated intravascular coagulation; HCO, hydrocortisone; HCQ, Hydroxychloroquine; ICU, intensive care unit; IFN, interferon *β*-1a; IMV, invasive mechanical ventilation; IV, intravenous; IVIG, intravenous immunoglobulins; LPV/r, Lopinavir-Ritonavir; MP, methylprednisolone; NA, not available; NLR, neutrophil-to-lymphocyte ratio; rtPA, recombinant tissue plasminogen activator; SOC, standard of care; SOFA, sequential organ failure assessment; UFH, unfractionated heparin; UFV, Umifenovir; VTE, venous thromboembolism.

^a^Multicenter: France, Ireland, Netherlands, Portugal, and UK, Australia, New Zealand, Canada, USA.

^b^Multicenter: Denmark, India, Sweden, and Switzerland.

^c^Multicenter: Canada, USA, France, Germany, Ireland, Netherlands, Portugal, UK, Saudi Arabia, Australia, New Zealand.

^d^Multicenter: UK, Netherlands, Australia, New Zealand, Ireland, Saudi Arabia.

^e^Multicenter: Netherlands, Germany, France, Belgium, Russia, Brazil, Peru, Mexico, and South Africa.

^f^Multicenter: France, Japan, Spain, the UK, and the USA.

^g^Multicenter: Argentina, Brazil, Mexico, and the USA.

^h^Multicenter: Argentina, Brazil, Canada, Chile, France, Germany, Israel, Italy, Japan, Russia, and Spain.

^i^Multicenter: Australia, Canada, the UK, and the US.

^j^Multicenter: UK, USA, Canada, Brazil, Ireland, Netherlands, Australia, Nepal, Saudi Arabia, and Mexico.

^1^Critically ill COVID-19 patients; patients admitted to an adult ICU or area of the hospital where critically ill patients routinely receive treatment, patients receiving invasive or noninvasive mechanical ventilation, patients receiving continuous intravenous vasoactive medications or other criteria with justification presented in the individual study to designate patients as critically ill.

^2^Defined as death or persistent dependency on mechanical ventilation or high-flow oxygen therapy).

^3^Composite of venous or arterial thrombosis, treatment with extracorporeal membrane oxygenation, or mortality within 30 days.

**Table 2:** Included studies outcomes related to the meta-analysis.

Group/Author	Interv. size	Control size	ICU mortality/interv.	ICU mortality/control	Hospital mortality/interv.	Hospital mortality/control	28 d mortality/intervention	28 d mortality/control
*Steroid*								
REMAP-CAP (HCO fixed dose), *n* (%)	137	101	—	—	41 (29.9)	33 (32.7)	—	—
REMAP-CAP (HCO shock dose), *n* (%)	141	101	—	—	37 (26.2)	33 (32.7)	—	—
CoDEX (DEX), *n* (%)	151	148	85 (56.3)	91 (61.5)	—	—	85 (56.3)	91 (61.5)
Dequin et al. (HCO), *n* (%)	76	73	11 (14.5)	20 (27.4)	—	—	11 (14.5)	20 (27.4)
COVID STEROID 1 (HCO), *n* (%)	16	14	—	—	—	—	6 (37.5)	2 (14.3)
COVID STEROID 2 (DEX), *n* (%)	491	480	—	—	—	—	133 (27.1)	155 (32.3)
Maskin et al. (DEX), *n* (%)	49	49	21 (42.9)	24 (49)	22 (44.9)	24 (49)	20 (40.8)	19 (38.8)
COVIDICUS (DEX), *n* (%)	270	276	—	—	—	—	69 (25.6)	68 (24.6)
Soliman et al. (DEX), *n* (%)	30	30	—	—	13 (43.3)	5 (16.7)	—	—

*Antiviral*								
Davoudi-Monfared et al. (IFN *β*-1a), *n* (%)	42	39	8 (19.1)	14 (35.9)	8 (19.0)	16 (41.0)	8 (19.0)	17 (43.6)
REMAP-CAP (LPV/r), *n* (%)	255	362	—	—	88 (34.5)	106 (29.3)	—	—
REMAP-CAP (HCQ), *n* (%)	50	362	—	—	17 (34.0)	106 (29.3)	—	—
REMAP-CAP (HCQ + LPV/r), *n* (%)	27	362	—	—	13 (48.1)	106 (29.3)	—	—
Alavi darazam et al. (UFV + LPV/r + HCQ + IFN *β*-1a), *n* (%)	51	50	—	—	17 (33.3)	19 (38.0)	—	—
Réa-Neto et al. (CQ/HCQ), *n* (%)	53	52	—	—	—	—	16 (30.2)	10 (19.2)

*Immunomodulators*								
PANAMO (Vilobelimab), *n* (%)	15	15	—	—	—	—	2 (13.3)	4 (26.7)
Fakharian et al. (Adalimumab), *n* (%)	34	34	—	—	4 (11.8)	4 (11.8)	—	—
Rashad et al. (Tocilizumab), *n* (%)	46	63	—	—	32 (69.6)	33 (52.4)	—	—
REMAP-CAP (Tocilizumab), *n* (%)	350	397	—	—	98 (28.0)	142 (35.8)	—	—
REMAP-CAP (Sarilumab), *n* (%)	45	397	—	—	10 (22.2)	142 (35.8)	—	—
COV-BARRIER (Baricitinib), *n* (%)	51	50	—	—	—	—	20 (39.2)	29 (58.0)
Lescure et al. (Sarilumab 400 mg), *n* (%)	173	84	—	—	—	—	14 (8.1)	7 (8.3)
Lescure et al. (Sarilumab 200 mg), *n* (%)	159	84	—	—	—	—	16 (10.1)	7 (8.3)
PANAMO phase 3 (Vilobelimab), *n* (%)	177	191	—	—	—	—	54 (30.5)	77 (40.3)
Atmowihardjo et al. (Imatinib), *n* (%)	33	33	—	—	—	—	5 (15.2)	6 (18.2)
CORIMUNO-19 (Tocilizumab), *n* (%)	49	43	—	—	12 (24.5)	13 (30.2)	8 (16.3)	10 (23.3)
CORIMUNO-19 (Sarilumab), *n* (%)	48	33	—	—	14 (29.2)	13 (39.4)	14 (29.2)	11 (33.3)
ESCAPE (Anakinra), *n* (%)	60	42	—	—	—	—	20 (33.3)	14 (33.3)
Kharazmi et al. (Anakinra), *n* (%)	15	15	—	—	5 (33.3)	7 (46.7)	—	—
Rein et al. (Ruxolitinib 15 mg), *n* (%)	77	47	—	—	—	—	39 (50.6)	33 (70.2)
Rein et al. (Ruxolitinib 5 mg), *n* (%)	87	47	—	—	—	—	45 (51.7)	33 (70.2)
Annane et al. (Ravulizumab), *n* (%)	135	66	—	—	—	—	55 (40.7)	25 (37.9)

*Plasma therapies*								
Rasheed et al. (Conv. Plasma), *n* (%)	21	28	1 (4.8)	8 (28.6)	1 (4.8)	8 (28.6)	—	—
Faqihi et al. (TPE), *n* (%)	43	44	—	—	—	—	9 (20.9)	15 (34.1)
Ali et al. (C-IVIG 0.15 g/kg), *n* (%)	10	10	—	—	—	—	2 (20.0)	6 (60)
Ali et al. (C-IVIG 0.20 g/kg), *n* (%)	10	10	—	—	—	—	3 (30.0)	6 (60)
Ali et al. (C-IVIG 0.25 g/kg), *n* (%)	10	10	—	—	—	—	1 (10.0)	6 (60)
Ali et al. (C-IVIG 0.30 g/kg), *n* (%)	10	10	—	—	—	—	4 (40.0)	6 (60)
Bennett-Guerrero et al. (Conv. Plasma), *n* (%)	58	14	—	—	—	—	14 (24.1)	4 (28.6)
REMAP-CAP (Conv. plasma), *n* (%)	1075	904	—	—	401 (37.3)	557 (61.6)	352 (32.7)	300 (33.2)
ICAR (C-IVIG 2 g/kg), *n* (%)	69	77	—	—	—	—	24 (34.8)	20 (26.0)

*Anti-coagulation*								
HESACOVID (Therap. Enoxaparin), *n* (%)	10	10	—	—	2 (20.0)	5 (50.0)	3 (30.0)	1 (10.0)
INSPIRATION (Interm. Enoxaparin), *n* (%)	276	286	—	—	119 (43.1)	117 (40.9)	—	—
REMAP-CAP (Therap. Heparin), *n* (%)	534	564	—	—	199 (37.3)	200 (35.5)	—	—
Barrett et al. (tPA), *n* (%)	19	17	—	—	4 (21.1)	7 (41.2)	4 (21.1)	5 (29.4)
Rashidi et al. (rtPA), *n* (%)	5	5	—	—	5 (100.0)	4 (80.0)	—	—
Rashidi et al. (Theraputic UFH), *n* (%)	5	5	—	—	2 (40.0)	4 (80.0)	—	—
Swiss COVID-HEP (Therap. Enoxaparin), *n* (%)	79	80	—	—	3 (3.8)	4 (5.0)	3 (3.8)	4 (5.0)
COVID-PACT (Theraputic UFH OR enoxaparin), *n* (%)	191	191	—	—	36 (18.8)	32 (16.8)	—	—
Garofalo et al. (Bivalirudin), *n* (%)	29	29	8 (27.6)	10 (34.5)	—	—	8 (27.6)	10 (34.5)

Abbreviations: Conv.; convalescent, CQ; Chloroquine, DEX; Dexamethasone, HCO; hydrocortisone, HCQ; Hydroxychloroquine, ICU; intensive care unit, IFN; interferon *β*-1a, Interv.; intervention, IVIG; intravenous immunoglobulins, LPV/r; Lopinavir-Ritonavir, rtPA; recombinant tissue plasminogen activator, TPE; therapeutic plasma exchange, UFV; Umifenovir.

## Data Availability

Data could be extracted from tables and figures in the manuscript.

## Supporting Information

The supporting information file contains the search strategy for databases, assessment of publication bias using funnel plot diagrams and Egger's regression test, as well as detailed forest plot diagrams for ICU, hospital, 28-day, and 90-day mortality for each group.
