# Supplementary material for: Efficacy of COVID-19 Treatments in Intensive Care Unit: A Systematic Review and Meta-Analysis of Randomized Controlled Trials
Source: Crit Care Res Pract. 2024 Nov 27;2024:2973795. doi: 10.1155/ccrp/2973795 (PMC11617054; doi:10.1155/ccrp/2973795)
Supplement: Supporting Information — Additional supporting information can be found online in the Supporting Information section. [file 2973795.f1.pdf]

Supplementary materials:

Efficacy of COVID-19 treatment in Intensive Care Unit: Systematic review and Meta-Analysis of Randomized Controlled Trials

Mahmoud Alwakeel M.D[1], Francois Abi Fadel M.D[1], Abdelrahman Nanah[2], Yan Wang[3], Mohamed K. A. Awad[4], Fatima Abdeljaleel[2], Mohammed Obeidat[2], Talha Saleem M.D[1], Saira Afzal M.D[5], Dina Alayan M.D[2], Mary Pat Harnegie[6], Xiaofeng Wang Ph.D[7] , Abhijit Duggal M.D[1], Peng Zhang M.D[1]

[1] Department of Pulmonary & Critical Care Medicine, Respiratory Institute, Cleveland Clinic, Cleveland, Ohio, USA

[2] Cleveland Clinic Fairview Hospital, Cleveland, Ohio, USA

[3] Department of Anesthesiology, Boston Medical Center, Boston, Massachusetts, USA

[4] Department of Pulmonary, Critical care and Allergy, University of Alabama, Birmingham, Alabama, USA

[5] Neurology Department, Cleveland Clinic Florida, Weston, Florida, USA Department of Internal Medicine, Cleveland

[6] Cleveland Clinic Alumni Library, Cleveland, Ohio, USA

[7] Qualitative Health Sciences, Cleveland Clinic, Cleveland, Ohio, USA

Corresponding author information:

Mahmoud Alwakeel MD

Department of Pulmonary and Critical Care, Cleveland Clinic, 9500 Euclid Ave, mail code A90, Cleveland, Ohio 44195.

E-mail: alwakem@ccf.org

Table of Contents:

Databases Search Strategy: 3
Publication Bias Assessment (Funnel Plot Diagram): 8
Supplementary Figure 1: Steroid group 8
Supplementary Figure 2: Antiviral Group: 9
Supplementary Figure 3: Immunomodulators Group: 10
Supplementary Figure 4: Plasma Therapies Group: 11
Supplementary Figure 5: Anti-coagulation Group: 12
Meta-analysis Forest Plot for ICU, Hospital, 28-days and 90-days Mortality: 13
Supplementary Figure 6: Association Between Antiviral Agents and Mortality: 13
Supplementary Figure 7: Association Between Steroids and Mortality: 14
Supplementary Figure 8: Association Between Immunomodulators and Mortality: 15
Supplementary Figure 9: Association Between Plasma Therapies and Mortality: 16
Supplementary Figure 10: Association Between Anti-coagulation and Mortality: 17
Supplementary Figure 11: PRISMA Checklist 18

Databases Search Strategy:

P= Critically ill patients
I= Covid 19
C= Randomized Controlled Trials
O=Identify Effective Pharmacological treatment
Restricted to adults and Covid period (2019- 4/9/21)
Updated 9/30/2021- all databases

Updated 4/28/2023-all databases

Ovid Medline (Ovid interface)- 2019-4/9/21
Updated 9/30/21

1 exp Coronavirus
2 exp Coronavirus Infections/
3 (coronavirus* or corona virus* or OC43 or NL63 or 229E or HKU1 or HCoV* or ncov* or covid* or sarscov* or sarscov* or Sars-coronavirus* or Severe Acute Respiratory Syndrome Coronavirus*).mp.
4 (or/1-3) and ((20191* or 202*).dp. or (20190101:20301231).ep.
5 ((pneumonia or covid* or coronavirus* or corona virus* or ncov* or 2019-ncov or sars*).mp. or exp pneumonia/) and Wuhan.mp.
6 (2019-ncov or ncov19 or ncov-19 or sars-cov2 or sars-cov-2 or sarscov2 or sarscov-2 or Sarscoronavirus2 or Sars-coronavirus-2 or coronavirus-19 or covid19 or covid-19 or covid 2019 or "2019-novel Cov" or ((novel or new or nouveau) adj2 (CoV or nCoV or covid or coronavirus* or corona virus or Pandemi*2)) or (coronavirus* and pneumonia)).mp.
7 COVID-19.rx,px,ox. or severe acute respiratory syndrome coronavirus 2.os.
8 or/5-7
9 4 not (SARS or SARS-CoV or MERS or MERS-CoV or Middle East respiratory syndrome or camel* or dromedar* or equine or coronary or coronal or covidence* or covidien or influenza virus or HIV or bovine or calves or TGEV or feline or porcine or erinaceus or BCoV or PED or PEDV or PDCoV or FIPV or FCoV or canine or CCov or zoonotic or avian influenza or H1N1 or H5N1 or H5N6 or IBV or murine corona*).mp.
10 8 and (SARS or SARS-CoV or MERS or MERS-CoV or Middle East respiratory syndrome or camel* or dromedar* or equine or coronary or coronal or covidence* or covidien or influenza virus or HIV or bovine or calves or TGEV or feline or porcine or erinaceus or BCoV or PED or PEDV or PDCoV or FIPV or FCoV or canine or CCov or zoonotic or avian influenza or H1N1 or H5N1 or H5N6 or IBV or murine corona*).mp.
11 or/8-10
12 11 and 20191201:20301231.dt.
13 "randomized controlled trial".pt. OR "controlled clinical trial".pt. OR randomized.ti,ab. OR placebo.ti,ab. OR "drug therapy"/OR randomly.ti,ab. OR trial.ti,ab. OR groups.ti,ab.
14 exp Critical Care/
15 intensive care.mp.
16 Intensive Care Units/
17 exp Respiration, Artificial/

18 mechanical ventila*.mp. or (Intubat* or ventilat*).ti,ab.
19 exp Vasoconstrictor Agents/ or "hypertensive factor".mp.
20 inotropes.mp.
21 ((critical* or intensive) adj3 (Ill* or care or therapy)).mp.
22 or/14-21
23 12 and 13 and 22
24 limit 23 to English language
25 (Pediatric* or paediatric* or child* or adolescen* or bab* or infant* or neonat*).ti,ab.
26 24 NOT 25

Ovid Embase (Ovid interface)- 1/1/2019-4/9/2021
Updated 9/30/21

1 exp Coronavirus/
2 exp Coronavirus Infections/
3 (coronavirus* or corona virus* or OC43 or NL63 or 229E or HKU1 or HCoV* or ncov* or covid* or sars-cov* or sarscov* or Sars-coronavirus* or Severe Acute Respiratory Syndrome Coronavirus*).mp.
4 (or/1-3) and (20190101:20301231).dc.
5 4 not (SARS or SARS-CoV or MERS or MERS-CoV or Middle East respiratory syndrome or camel* or CCov or zoonotic or avian influenza or H1N1 or H5N1 or H5N6 or IBV or murine corona*).mp.
6 ((pneumonia or covid* or coronavirus* or corona virus* or ncov* or 2019-ncov or sars*).mp. or exp pneumonia/) and Wuhan.mp.
7 (coronavirus disease 2019 dromedar* or equine or coronary or coronal or covidence* or covidien or influenza virus or HIV or bovine or calves or TGEV or feline or porcine or BCoV or PED or PEDV or PDCoV or FIPV or FCoV or SADS-CoV or canine or or 2019-ncov or ncov19 or ncov-19 or 2019-novel CoV or severe acute respiratory syndrome coronavirus 2 or sars-cov2 or sars-cov-2 or sarscov2 or sarscov-2 or Sars-coronavirus2 or Sars-coronavirus-2 or SARS-like coronavirus* or coronavirus-19 or covid19 or covid-19 or covid 2019 or ((novel or new or nouveau) adj2 (CoV or nCoV or covid or coronavirus* or corona virus or Pandemi*2)) or ((covid or covid19 or covid-19) and pand) or (coronavirus* and emic*2 pneumonia)).mp.
8 (coronavirus disease 2019 or severe acute respiratory syndrome coronavirus 2).sh,dj.
9 (or/6-8) and (20191201:20301231).dc.
10 5 or 9
11 exp 'randomized controlled trial'/ OR exp 'controlled clinical trial'/ OR randomized.ti,ab. OR placebo.ti,ab. OR 'drug therapy'/ OR randomly.ti,ab. OR trial.ti,ab. OR groups.ti,ab.
12 exp critically ill patient/
13 exp intensive care/
14 icu.mp. or exp intensive care unit/
15 mechanical ventilation.mp. or exp artificial ventilation/ or (intubat* or ventilat*).ti,ab.
16 exp hypertensive factor/
17 exp vasoconstrictor agent/
18 inotrope.mp. or exp inotropic agent/
19 ((critical* or intensive) adj3 (ill* or unit or therap*)).mp.
20 OR/12-19
21 10 and 11 and 20

22 limit 21 to English language
23 (Pediatric* or paediatric* or child* or adolescen* or bab* or infant* or neonat*).ti,ab.
24 22 not 23

<u>CINAHL (Ebsco Interface)- 1974-4/9/21</u>
<u>Updated 9/30/21</u>

coronavirus* or corona virus* or OC43 or NL63 or 229E or HKU1 or HCoV* or ncov* or covid* or sarscov* or sarscov* or Sars-coronavirus* or Severe Acute Respiratory Syndrome Coronavirus*) OR (MH "COVID-19") OR "covid 19" OR (MH "Coronavirus") OR "coronavirus" OR (MH "Coronavirus Infections") OR (MH "Middle East Respiratory Syndrome Coronavirus") OR ((pneumonia or covid* or coronavirus* or corona virus* or ncov* or 2019-ncov or sars*) or pneumonia) and Wuhan) OR (2019-ncov or ncov19 or ncov-19 or sars-cov2 or sars-cov-2 or sarscov2 or sarscov-2 or Sarscoronavirus2 or Sars-coronavirus-2 or coronavirus-19 or covid19 or covid-19 or covid 2019 or "2019-novel Cov" or ((novel or new or nouveau) N2 (CoV or nCoV or covid or coronavirus* or corona virus or Pandemi*2)) or (coronavirus* and pneumonia))

AND TI (random* or placebo* or groups* or trial*) OR AB (random* or placebo* or groups* or trial*) OR (MH "Randomized Controlled Trials") OR "randomized controlled trials" OR (MH "Clinical Trials")

AND

(MH "Critical Care") OR "intensive care" OR (MH "Intensive Care Unit") OR (MH "Respiration, Artificial") OR "artificial respiration" OR mechanical ventilation OR AB ((intubat* or ventilat*)) AND TI ((intubat* or ventilat*))

NOT

camel* or dromedar* or equine or coronary or coronal or covidence* or covidien or influenza virus or HIV or bovine or calves or TGEV or feline or porcine or erinaceus or BCoV or PED or PEDV or PDCoV or FIPV or FCoV or canine or CCov or zoonotic or avian influenza or H1N1 or H5N1 or H5N6 or IBV or murine corona*

NOT

T(child* or infant or bab* or neonat* or adolescen* or fetal or fetus or pediatric* or paediatric*)

<u>Cochrane Library (Wiley interface)- 1974-4/9/21</u>
<u>Updated 9/30/21</u>

coronavirus* or corona virus* or OC43 or NL63 or 229E or HKU1 or HCoV* or ncov* or covid* or sarscov* or sarscov* or Sars-coronavirus* or Severe Acute Respiratory Syndrome OR ((pneumonia or covid* or coronavirus* or corona virus* or ncov* or 2019*ncov or sars*) and Wuhan) Or ((2019*ncov or ncov19 or ncov*19 or sars*cov2 or sars*cov*2 or sarscov2 or sarscov*2 or Sarscoronavirus2 or Sars*coronavirus*2 or coronavirus*19 or covid19 or covid*19 or covid 2019 or 2019*novel Cov or novel or new or nouveau) NEAR/2 (CoV or nCoV or covid or coronavirus* or corona virus or Pandemi*2 OR coronavirus* and pneumonia))

AND

controlled clinical trial OR random* OR placebo* OR randomi?ed controlled trial. OR trial OR groups OR MeSH descriptor: [Drug Therapy] explode all trees

AND

((critical* or intensive) NEAR/3 (Ill* or care or therapy)) OR MeSH descriptor: [Intensive Care Units] explode all trees OR MeSH descriptor: [Critical Care] explode all trees OR MeSH descriptor: [Critical Illness] explode all trees OR MeSH descriptor: [Respiration, Artificial] explode all trees OR MeSH descriptor: [Respiration, Artificial] explode all trees OR MeSH descriptor: [Vasoconstrictor Agents] explode all trees OR Mechanical ventilat* or intubat* or ventilat* or "hypertensive factor" or inotrope*

NOT

porcine or erinaceus or BCoV or PED or PEDV or PDCoV or FIPV or FCoV or canine or CCov or zoonotic or avian influenza or H1N1 or H5N1 or H5N6 or IBV or murine corona*
NOT
(child* or infant or bab* or neonat* or adolescen* or fetal or fetus or pediatric* or paediatric*)
Limit to 1/1/19-12/31/21

Web of Science (1974-4/9/2021)
Updated 9/30/21

TS=((coronavirus* or corona virus* or OC43 or NL63 or 229E or HKU1 or HCoV* or ncov* or covid* or sars-cov* or sarscov* or Sars-coronavirus* or Severe Acute Respiratory Syndrome Coronavirus*) OR TS=((pneumonia or covid* or coronavirus* or corona virus* or ncov* or 2019-ncov or sars*) and Wuhan) OR TS=((coronavirus disease 2019 or 2019-ncov or ncov19 or ncov-19 or 2019-novel CoV or severe acute respiratory syndrome coronavirus 2 or sars-cov2 or sars-cov-2 or sarscov2 or sarscov-2 or Sars-coronavirus2 or Sars-coronavirus-2 or SARS-like coronavirus* or coronavirus-19 or covid19 or covid19 or covid 2019 or novel or new or nouveau)) OR TS= ((CoV or nCoV or covid or coronavirus* or corona virus or Pandemi*2)) or TS=((covid or covid19 or covid-19) and pandemic*2) or TS=(coronavirus* and pneumonia)
AND
TS=("randomized controlled trial" OR "controlled clinical trial") OR TI=(randomi?ed OR placebo OR " drug therapy" OR random* OR trial OR groups) OR AB=(randomi?ed OR placebo OR "drug therapy" OR random* OR trial OR groups)
AND
TS=((critical* or intensive) NEAR/3 (Ill* or care or therapy)) OR
TI=(intubat* or ventilat*) or AB=(intubat* or ventilat*) or TS=("hypertensive factor" or vasoconstrictor agent* OR vasopressor* or inotrope*) OR TS=(artificial respirat*) or TS=(mechanical* ventilat*)
NOT
TI=(Pediatric* or paediatric* or child* or adolescen* or bab* or infant* or neonat*or fetal or fetal) or AB=(Pediatric* or paediatric* or child* or adolescen* or bab* or infant* or neonat*or fetal)
NOT
TS=(dromedar* or equine or coronary or coronal or covidence* or covidien or influenza virus or HIV or bovine or calves or TGEV or feline or porcine or BCoV or PED or PEDV or PDCoV or FIPV or FCoV or SADS-CoV or canine)
Limit to English, 2019-2021, exclude Medline, Case Reports, Editorials, Letters

Scopus (Elsevier interface)- 1974-4/9/21
Updated 9/30/21
TITLE-ABS-KEY (coronavirus* OR corona AND virus* OR oc43 OR nl63 OR 229e OR hku1 OR hcov* OR ncov* OR covid* OR sars-cov* OR sarscov* OR sars-coronavirus* OR severe AND acute AND respiratory AND syndrome AND coronavirus) OR TITLE-ABS-KEY-AUTH ((pneumonia OR covid* OR coronavirus* OR corona AND virus* OR ncov* OR 2019-ncov OR sars*) AND wuhan) OR TITLE-ABS-KEY (coronavirus AND disease 2019 OR 2019-ncov OR ncov19 OR ncov-19 OR 2019-novel AND cov OR severe AND acute AND respiratory AND syndrome AND coronavirus 2 OR sars-cov2 OR sars-cov-2 OR sarscov2 OR sarscov-2 OR sars-coronavirus2 OR sars-coronavirus-2 OR sars-like AND coronavirus* OR coronavirus-19 OR covid19 OR covid19 OR covid 2019 OR novel OR new OR nouveau) OR TITLE-ABS-KEY ((cov OR ncov OR covid OR coronavirus* OR corona AND virus OR pandemi*2)) OR TITLE-ABS-KEY ((covid19 OR covid-19) AND pandemic*2) OR TITLE-ABS-KEY (coronavirus* AND pneumonia)
AND
TITLE-ABS-KEY ("randomized controlled trial" OR "controlled clinical trial") OR TITLE-ABS (

randomi?ed OR placebo OR "drug therapy" OR random* OR trial OR groups)
AND
TITLE-ABS-KEY ((critical* OR intensive) W/3 (ill* OR care OR therapy)) OR TITLE-ABS (intubat* OR ventilat*) OR TITLE-ABS-KEY ("hypertensive factor" OR vasoconstrictor AND agent* OR vasopressor* OR inotrope*) OR TITLE-ABS-KEY (artificial AND respirat*) OR TITLE-ABS-KEY (mechanical* AND ventilat*)
NOT
TITLE-ABS (pediatric* OR paediatric* OR child* OR adolescen* OR bab* OR infant* OR neonat*or AND fetal OR fetal)
NOT

TITLE-ABS-KEY (dromedar* OR equine OR coronary OR coronal OR covidence* OR covidien OR influenza AND virus OR hiv OR bovine OR calves OR tgev OR feline OR porcine OR bcov OR ped OR pedv OR pdcov OR fipv OR fcov OR sads-cov OR canine)
NOT
TITLE-ABSTRACT(“case report” OR “case study”)
Limit to 2019-2021, English, Medicine & Medicine related classes, exclude letters, editors, & book chapters

Publication Bias Assessment (Funnel Plot Diagram):

Supplementary Figure 1: Steroid group

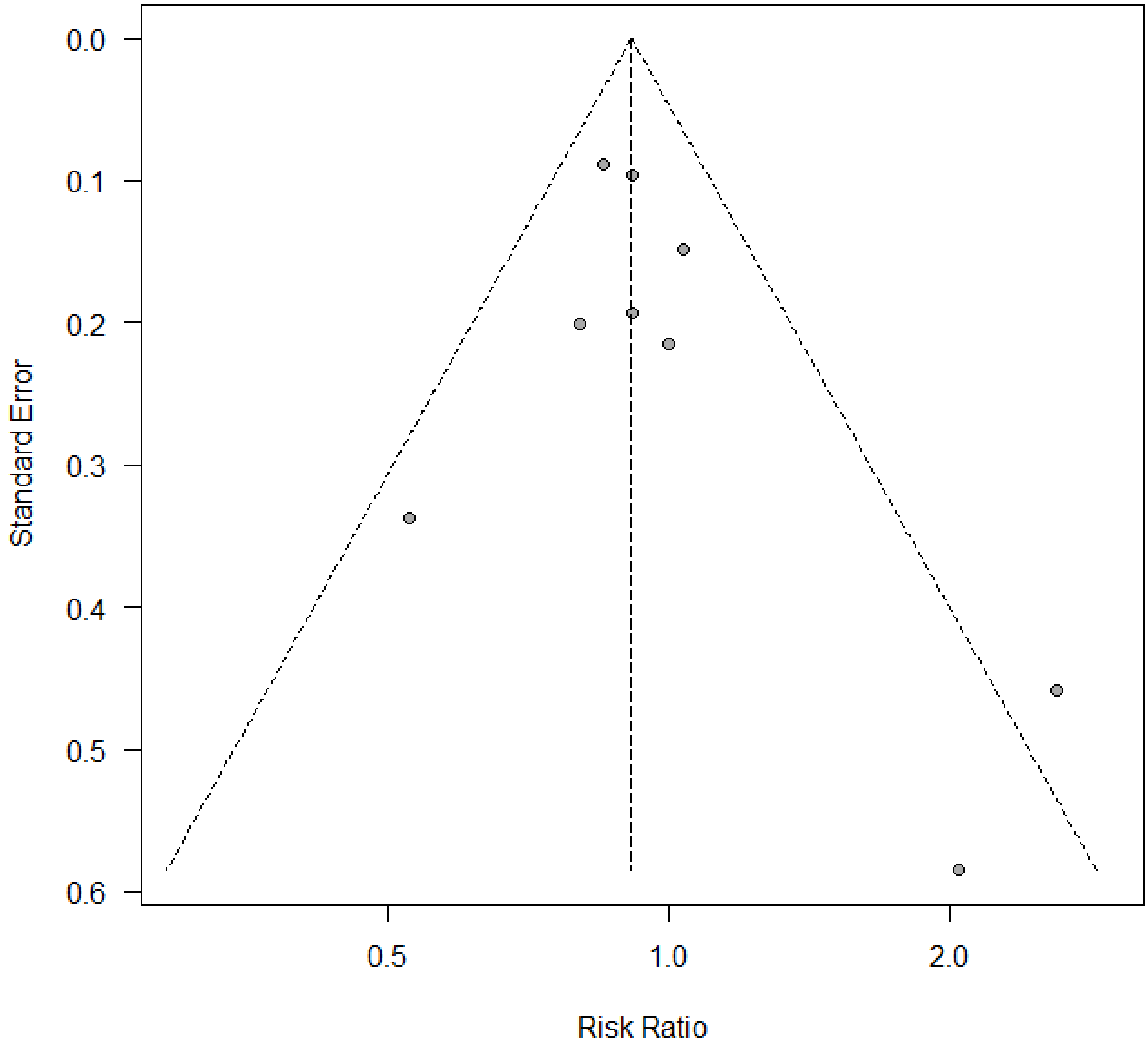

Funnel plot assessing publication bias in steroid group. Egger's test: Intercept = 0.95, CI 95% -0.91 to 2.81, p = 0.27

Supplementary Figure 2: Antiviral Group:

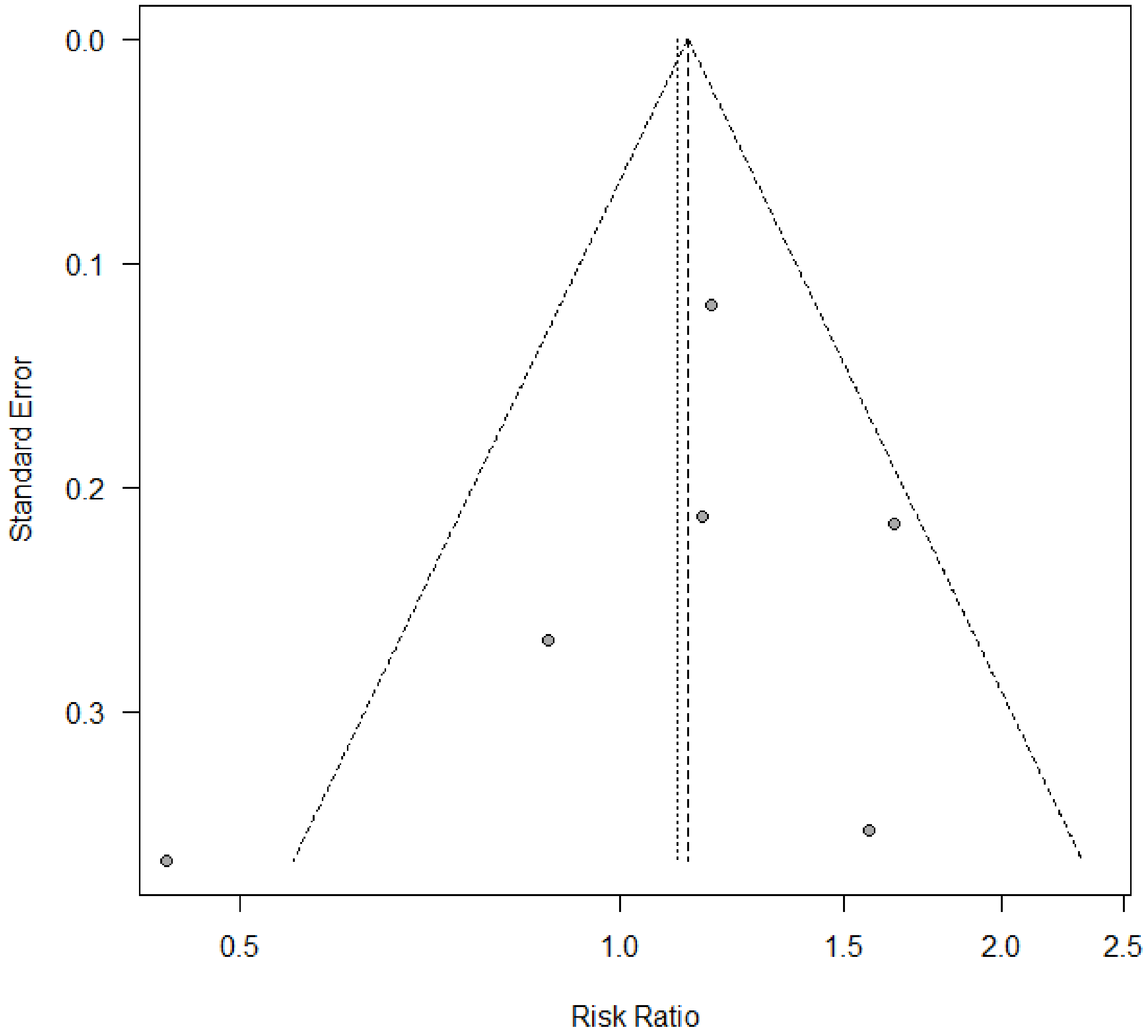

Funnel plot assessing publication bias in antiviral group. Egger's test: Intercept = -1.18, CI 95% -5.83 to 3.48, p = 0.27

Supplementary Figure 3: Immunomodulators Group:

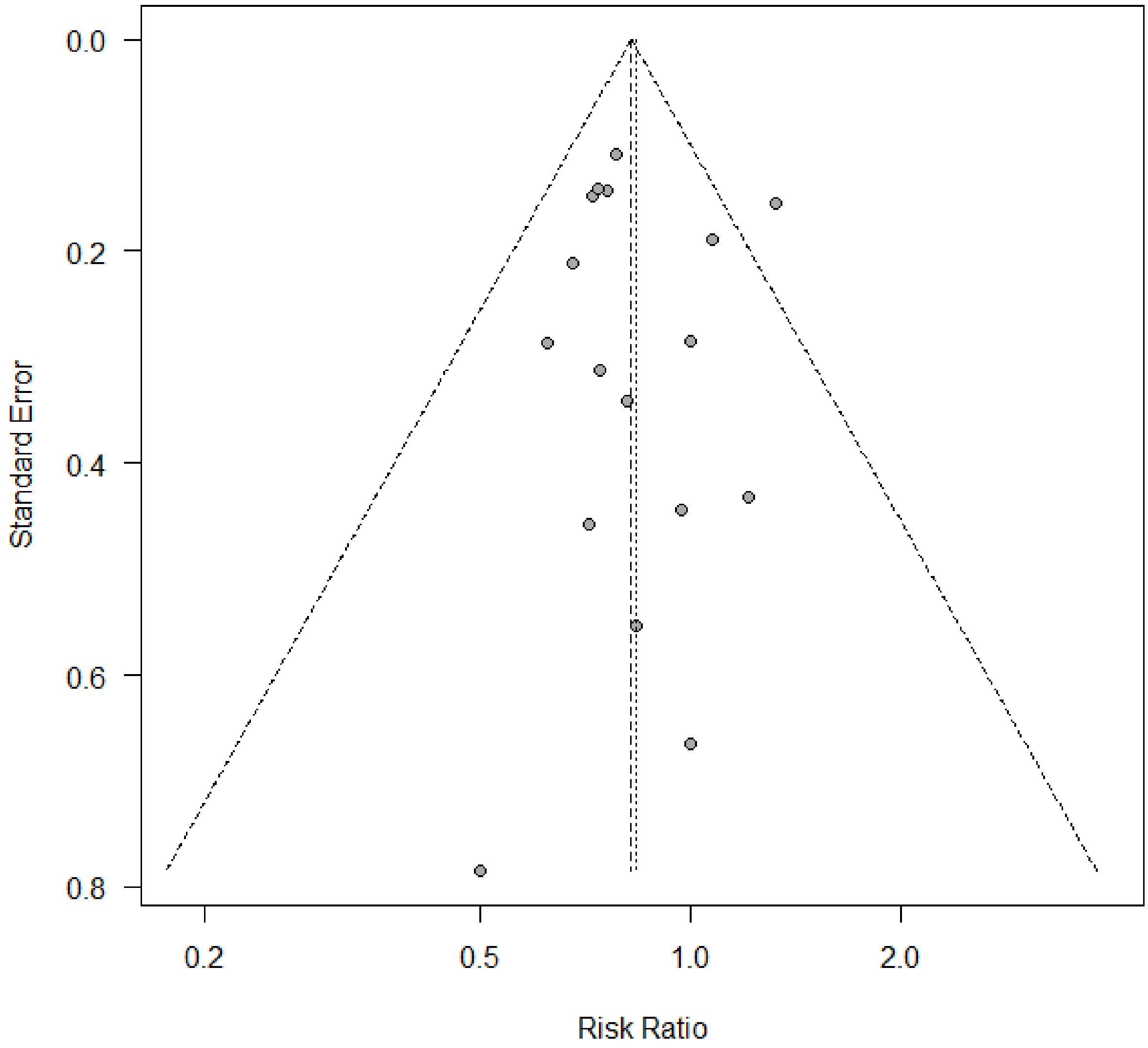

Funnel plot assessing publication bias in Immunomodulators group. Egger's test: Intercept = 0.07, CI 95% -1.08 to 1.22, p = 0.89

Supplementary Figure 4: Plasma Therapies Group:

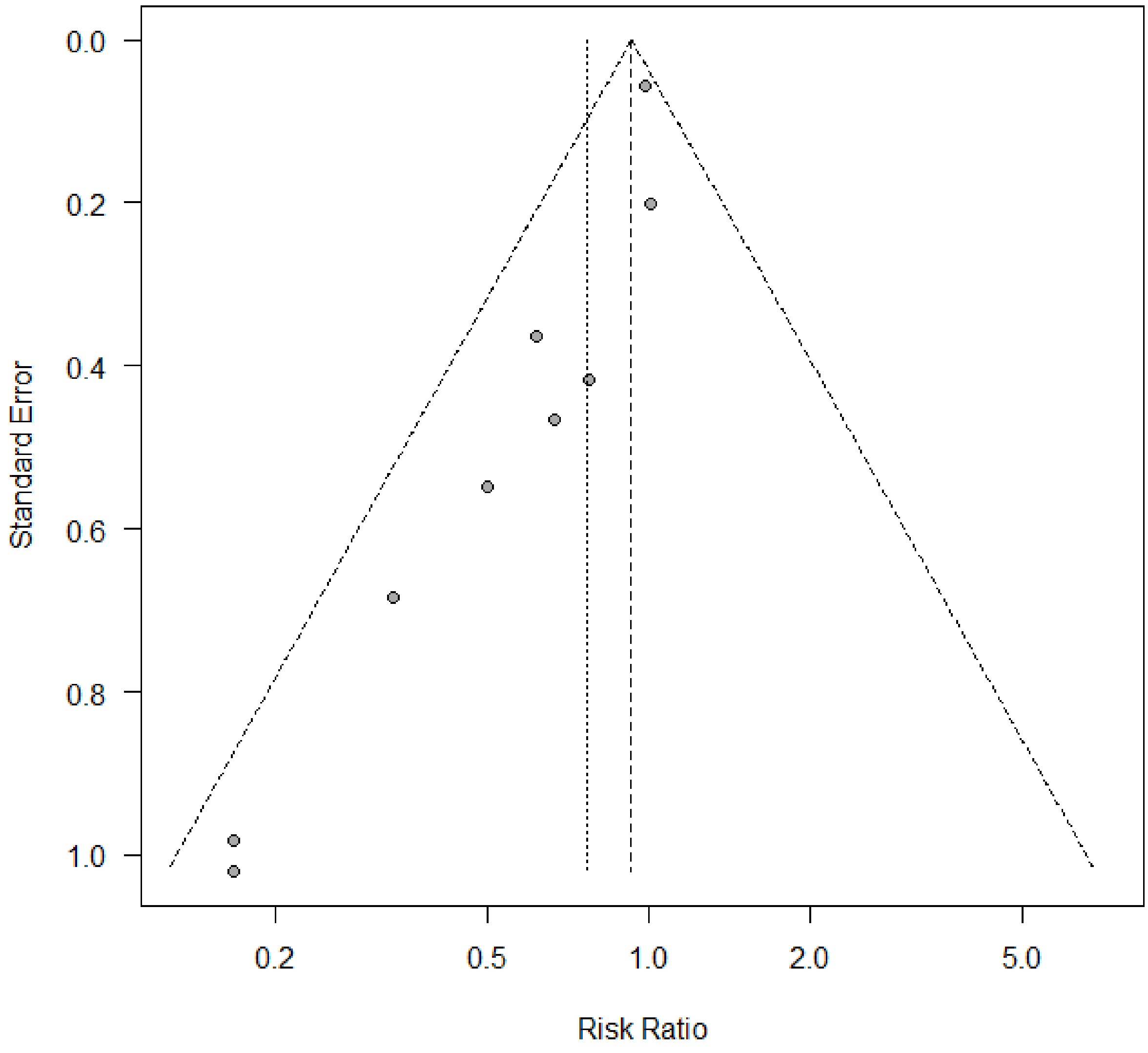

Funnel plot assessing publication bias in plasma therapies group. Egger's test: Intercept = -1.36, CI 95% -1.92 to -0.79, p < 0.01

Supplementary Figure 5: Anti-coagulation Group:

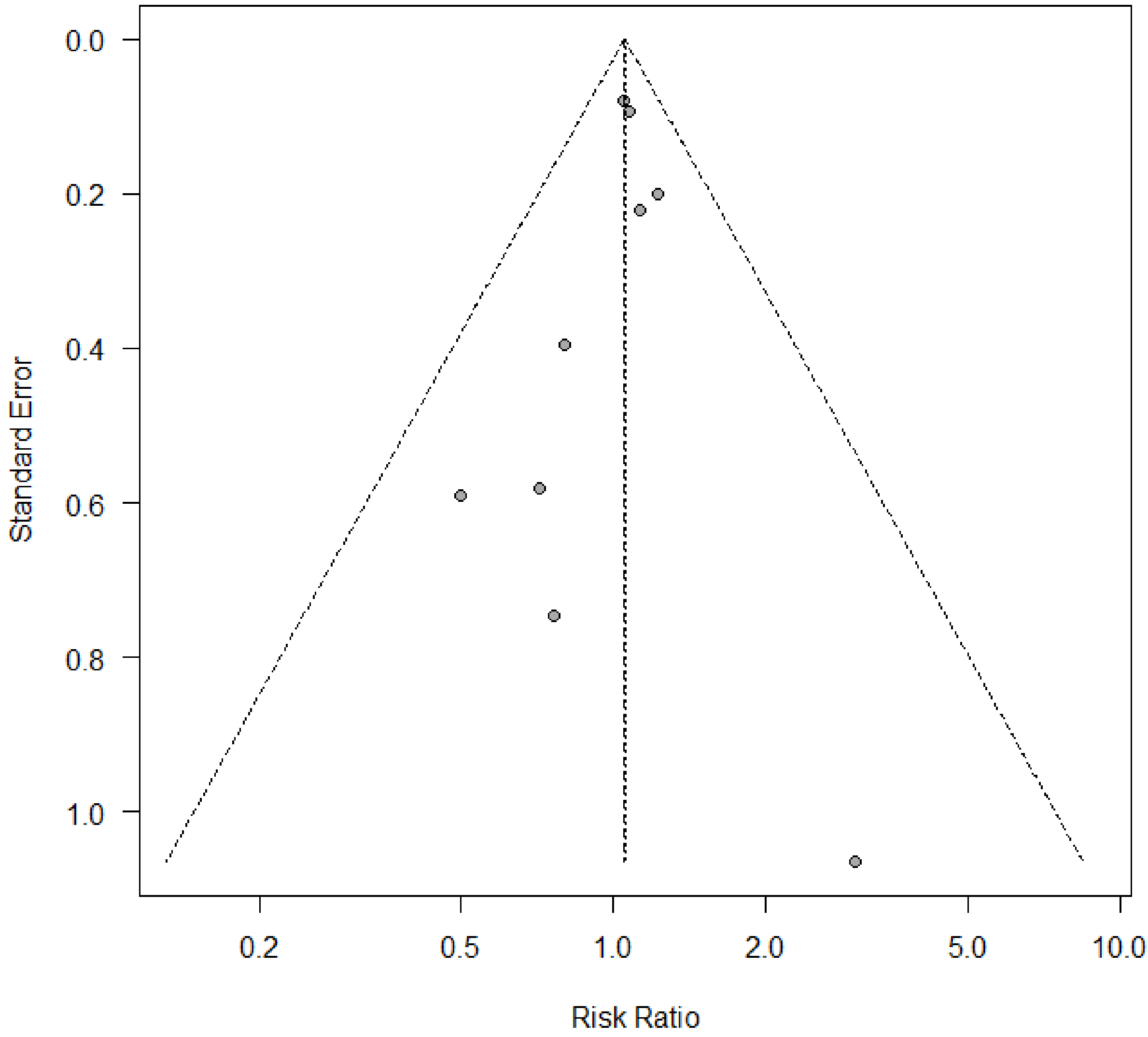

Funnel plot assessing publication bias in anti-coagulation group. Egger's test: Intercept = -0.27, CI 95% -1.13 to 0.59, p = 0.48

<u>Meta-analysis Forest Plot for ICU, Hospital, 28-days and 90-days Mortality:</u>

Supplementary Figure 6: Association Between Antiviral Agents and Mortality:

(a)

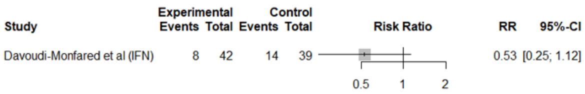

(b)

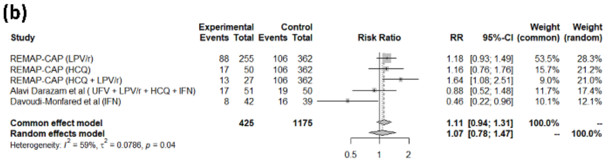

(c)

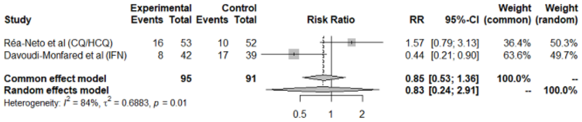

(d)

(a) ICU mortality, (b) Hospital mortality, (c) 28 days mortality, (d) 90 days mortality (no available data)

Supplementary Figure 7: Association Between Steroids and Mortality:

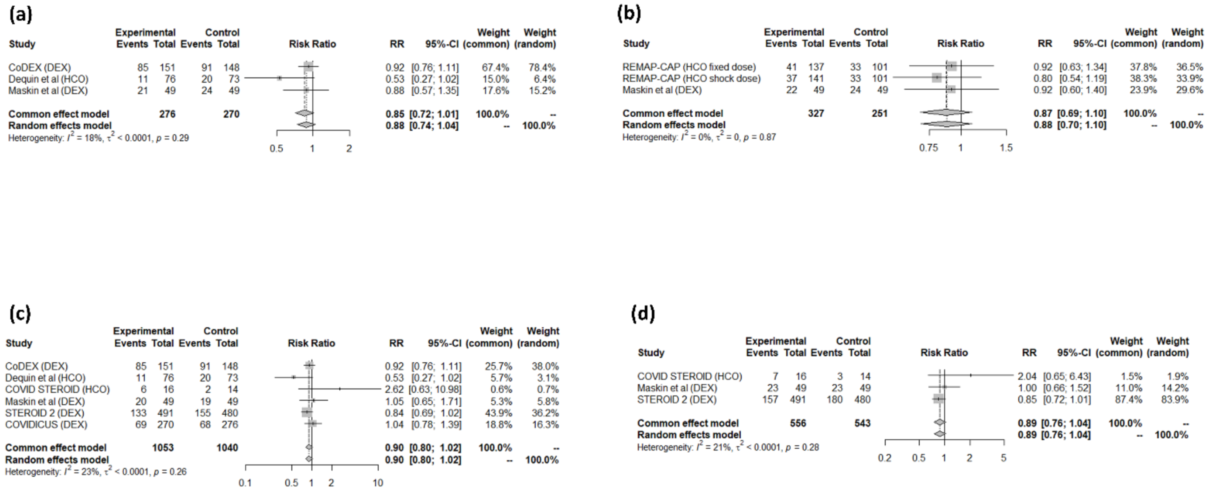

(a) ICU mortality, (b) Hospital mortality, (c) 28 days mortality, (d) 90 days mortality

Supplementary Figure 8: Association Between Immunomodulators and Mortality:

(a)

(b)

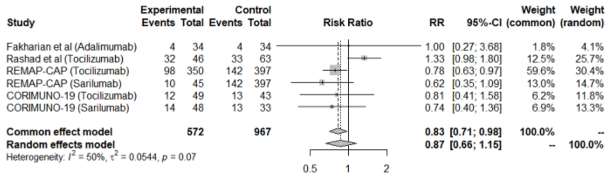

(c)

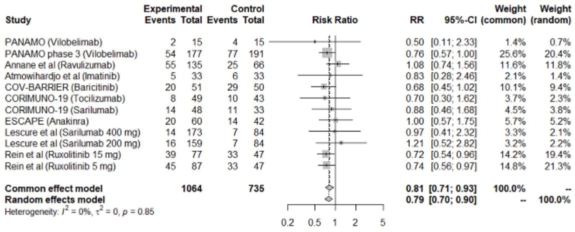

(d)

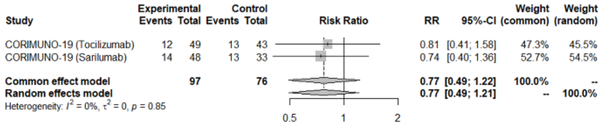

(a) ICU mortality (no available data), (b) Hospital mortality, (c) 28 days mortality, (d) 90 days mortality

Supplementary Figure 9: Association Between Plasma Therapies and Mortality:

(a)

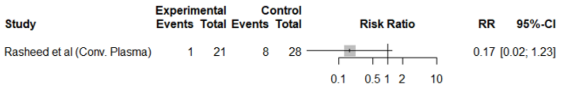

(b)

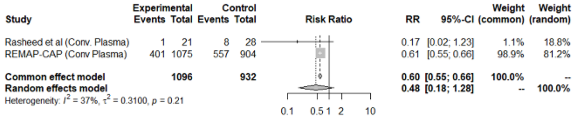

(c)

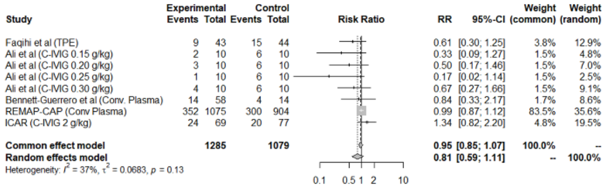

(d)

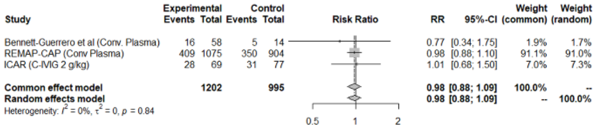

(a) ICU mortality, (b) Hospital mortality, (c) 28 days mortality, (d) 90 days mortality

Supplementary Figure 10: Association Between Anti-coagulation and Mortality:

(a)

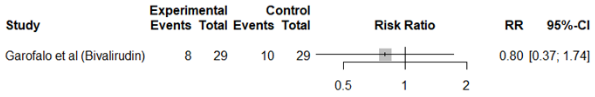

(b)

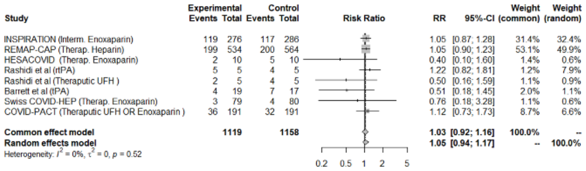

(c)

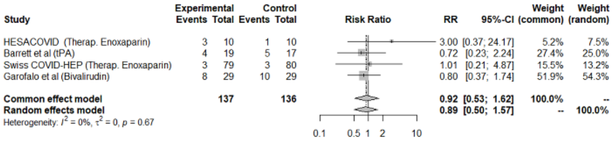

(d)

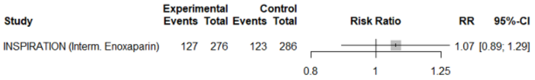

(a) ICU mortality, (b) Hospital mortality, (c) 28 days mortality, (d) 90 days mortality

Supplementary Figure 11: PRISMA Checklist

PRISMA 2020 Checklist

Section and Topic	Item #	Checklist item	Location where item is reported
**TITLE**			
Title	1	Identify the report as a systematic review.	P 1
**ABSTRACT**			
Abstract	2	See the PRISMA 2020 for Abstracts checklist.	P 2
**INTRODUCTION**			
Rationale	3	Describe the rationale for the review in the context of existing knowledge.	P 3
Objectives	4	Provide an explicit statement of the objective(s) or question(s) the review addresses.	P 3
**METHODS**			
Eligibility criteria	5	Specify the inclusion and exclusion criteria for the review and how studies were grouped for the syntheses.	P 3 - 4
Information sources	6	Specify all databases, registers, websites, organisations, reference lists and other sources searched or consulted to identify studies. Specify the date when each source was last searched or consulted.	P 4
Search strategy	7	Present the full search strategies for all databases, registers and websites, including any filters and limits used.	Suppl. P 3 - 7
Selection process	8	Specify the methods used to decide whether a study met the inclusion criteria of the review, including how many reviewers screened each record and each report retrieved, whether they worked independently, and if applicable, details of automation tools used in the process.	P 4
Data collection process	9	Specify the methods used to collect data from reports, including how many reviewers collected data from each report, whether they worked independently, any processes for obtaining or confirming data from study investigators, and if applicable, details of automation tools used in the process.	P 4 - 5
Data items	10a	List and define all outcomes for which data were sought. Specify whether all results that were compatible with each outcome domain in each study were sought (e.g. for all measures, time points, analyses), and if not, the methods used to decide which results to collect.	P 4 - 5
	10b	List and define all other variables for which data were sought (e.g. participant and intervention characteristics, funding sources). Describe any assumptions made about any missing or unclear information.	P 4 - 5
Study risk of bias assessment	11	Specify the methods used to assess risk of bias in the included studies, including details of the tool(s) used, how many reviewers assessed each study and whether they worked independently, and if applicable, details of automation tools used in the process.	P 5
Effect measures	12	Specify for each outcome the effect measure(s) (e.g. risk ratio, mean difference) used in the synthesis or presentation of results.	P 5
Synthesis methods	13a	Describe the processes used to decide which studies were eligible for each synthesis (e.g. tabulating the study intervention characteristics and comparing against the planned groups for each synthesis (item #5)).	P 5
	13b	Describe any methods required to prepare the data for presentation or synthesis, such as handling of missing summary statistics, or data conversions.	P 5
	13c	Describe any methods used to tabulate or visually display results of individual studies and syntheses.	P 5
	13d	Describe any methods used to synthesize results and provide a rationale for the choice(s). If meta-analysis was performed, describe the model(s), method(s) to identify the presence and extent of statistical heterogeneity, and software package(s) used.	P 5
	13e	Describe any methods used to explore possible causes of heterogeneity among study results (e.g. subgroup analysis, meta-regression).	P 5
	13f	Describe any sensitivity analyses conducted to assess robustness of the synthesized results.	P 5
Reporting bias assessment	14	Describe any methods used to assess risk of bias due to missing results in a synthesis (arising from reporting biases).	P 5
Certainty	15	Describe any methods used to assess certainty (or confidence) in the body of evidence for an outcome.	P 5

PRISMA 2020 Checklist

Section and Topic	Item #	Checklist item	Location where item is reported
assessment			
**RESULTS**			
Study selection	16a	Describe the results of the search and selection process, from the number of records identified in the search to the number of studies included in the review, ideally using a flow diagram.	P 6
	16b	Cite studies that might appear to meet the inclusion criteria, but which were excluded, and explain why they were excluded.	P 6
Study characteristics	17	Cite each included study and present its characteristics.	P 6, P 18-22
Risk of bias in studies	18	Present assessments of risk of bias for each included study.	P 7-8, P 28 - 30
Results of individual studies	19	For all outcomes, present, for each study: (a) summary statistics for each group (where appropriate) and (b) an effect estimate and its precision (e.g. confidence/credible interval), ideally using structured tables or plots.	P 7 – 8, P 21 – 22
Results of syntheses	20a	For each synthesis, briefly summarise the characteristics and risk of bias among contributing studies.	P 7 – 8, P 23 - 27
	20b	Present results of all statistical syntheses conducted. If meta-analysis was done, present for each the summary estimate and its precision (e.g. confidence/credible interval) and measures of statistical heterogeneity. If comparing groups, describe the direction of the effect.	P 7 – 8, P 23 – 27
	20c	Present results of all investigations of possible causes of heterogeneity among study results.	P 18 - 22
	20d	Present results of all sensitivity analyses conducted to assess the robustness of the synthesized results.	Supp. P 13 - 17
Reporting biases	21	Present assessments of risk of bias due to missing results (arising from reporting biases) for each synthesis assessed.	P 28 – 30, Supp. P 8 – 12
Certainty of evidence	22	Present assessments of certainty (or confidence) in the body of evidence for each outcome assessed.	P 7 - 8, P 21 - 27
**DISCUSSION**			
Discussion	23a	Provide a general interpretation of the results in the context of other evidence.	P 8
	23b	Discuss any limitations of the evidence included in the review.	P 11
	23c	Discuss any limitations of the review processes used.	P 11
	23d	Discuss implications of the results for practice, policy, and future research.	P 8 – 12
**OTHER INFORMATION**			
Registration and protocol	24a	Provide registration information for the review, including register name and registration number, or state that the review was not registered.	P 3
	24b	Indicate where the review protocol can be accessed, or state that a protocol was not prepared.	P 3
	24c	Describe and explain any amendments to information provided at registration or in the protocol.	NA
Support	25	Describe sources of financial or non-financial support for the review, and the role of the funders or sponsors in the review.	P 1
Competing interests	26	Declare any competing interests of review authors.	P 1
Availability of data, code and	27	Report which of the following are publicly available and where they can be found: template data collection forms; data extracted from included	P 18 - 22

PRISMA 2020 Checklist

Section and Topic	Item #	Checklist item	Location where item is reported
other materials		studies; data used for all analyses; analytic code; any other materials used in the review.	

From: Page MJ, McKenzie JE, Bossuyt PM, Boutron I, Hoffmann TC, Mulrow CD, et al. The PRISMA 2020 statement: an updated guideline for reporting systematic reviews. BMJ 2021;372:n71. doi: 10.1136/bmj.n71
